# Occupational Exposures and Skin Cancer: A Brief Report

**DOI:** 10.1111/srt.70107

**Published:** 2024-10-10

**Authors:** Aarushi K. Parikh, Isabella J. Tan, Bernard A. Cohen

**Affiliations:** ^1^ Department of Dermatology Rutgers Robert Wood Johnson Medical School New Brunswick New Jersey USA; ^2^ Department of Dermatology The Johns Hopkins Hospital Baltimore Maryland USA

Skin cancer is a major global public health issue, driven by environmental and occupational exposures. Incidence rates, including squamous cell carcinoma (SCC), basal cell carcinoma (BCC), melanoma, and rarer types like nail cancers, vary by region with significant disparities in mortality. Melanoma incidence in the United States has doubled from 1982 to 2011, with a further 31.5% rise from 2011 to 2019 [1]. Key risk factors include ultraviolet radiation (UVR) and chemical carcinogens, such as arsenic and polycyclic aromatic hydrocarbons, particularly in outdoor and industrial settings (Figure 1). This report reviews the literature on the distinct risk profiles of skin cancers, emphasizing the need for tailored management and preventive strategies based on environmental and occupational risks.

**FIGURE 1:**
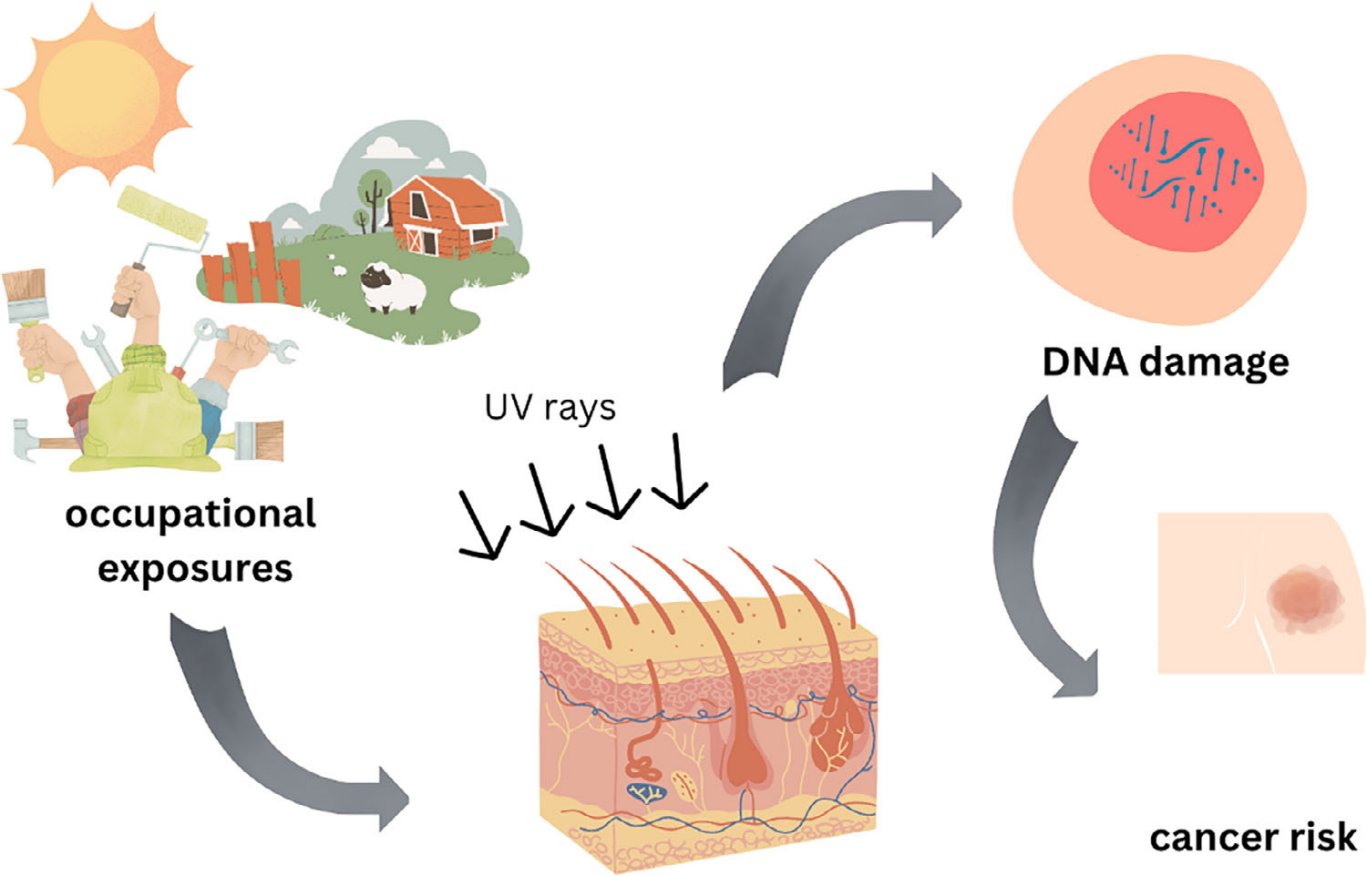
Pathway of enhanced skin cancer risk from occupational UV exposure.

In outdoor occupations such as agriculture, construction, and maritime industries, workers are at elevated risk for skin cancer due to chronic exposure to environmental carcinogens. In agriculture, pesticide exposure to carcinogenic chemicals like organochlorines and organophosphates, combined with extended UVR from sun exposure, contributes to an increased incidence of non‐melanoma skin cancers. Construction workers face significant UV exposure and are also exposed to chemical carcinogens such as solvents, tar, and asphalt, which have been shown to increase the risk for SCC and BCC [2]. Similarly, groundskeeping, gardening, and certain service‐related occupations were also found to face elevated risks for skin malignancy due to similar exposures [2]. Maritime workers experience intensified UVR due to water reflection, with UV exposures reaching 1975 J/m^2^, exceeding recommended daily limits and increasing melanoma risk [3].

Workers in industrial occupations are also vulnerable to UVR levels that surpass permissible limits, resulting in increased rates of skin disorders [4]. Additionally, long‐term exposure to metal arc welding is associated with a higher risk of BCC and actinic keratosis (AK) [5]. For offshore petroleum workers, who are exposed to UVR and other carcinogens, there is a notable increase in melanoma and non‐melanoma skin cancer (NMSC) risk [6]. Dermal exposure to crude oil or benzene also potentially contributes to increased skin cancer risk due to the absorption of polycyclic aromatic hydrocarbons [7].

Exposure to UVR and arsenic/radon in occupations such as mining leads to increased risk of NMSC in these settings. Regardless of exposure to solar radiation and skin type, miners have demonstrated increased NMSC cancer risk [8], emphasizing the need for enhanced ventilation systems to reduce radon levels, regular monitoring of radon concentrations, and the use of protective clothing.

Deployment‐related exposures also lead to a heightened risk of developing skin cancers. Melanoma and NMSC risk are particularly pronounced among service members, with the highest rates observed in the United States Air Force [9]. Contributing factors include extensive sun exposure in tropical and high‐altitude environments, inadequate sun protection, and exposure to burn pits [10].

SCC and BCC are primarily associated with chronic sun exposure, making outdoor workers, such as those in construction, agriculture, and landscaping, particularly vulnerable. Similarly, military personnel face heightened risk from extensive UV exposure. In the industrial, manufacturing, and chemical industries, workers encounter elevated NMSC risks due to exposure to hazardous chemicals and UVR. Each occupation presents unique UV and chemical exposure patterns that elevate the incidence of skin cancers, and access to protective gear and education are essential.

This report underscores the substantial impact of occupational exposures on skin cancer risk and the need for targeted clinical and preventive strategies. High‐risk occupations including agriculture, construction, maritime, and military service face increased skin cancer risks from exposure to UVR and chemical carcinogens. Effective protective measures and tailored interventions are essential. Future research should focus on refining exposure limits and enhancing management practices to address these risks.

## Ethics Statement

The authors have nothing to report.

## Conflicts of Interest

The authors declare no conflicts of interest.

## Data Availability

The data that support the findings of this study are available at https://pubmed.ncbi.nlm.nih.gov/.
